# Statistical Inference for Geometric Process with the Power Lindley Distribution

**DOI:** 10.3390/e20100723

**Published:** 2018-09-21

**Authors:** Cenker Bicer

**Affiliations:** Statistics Department, University of Kirikkale, 71450 Kirikkale, Turkey; cbicer@kku.edu.tr

**Keywords:** geometric process, maximum likelihood estimate, modified moment estimate, modified L-moment estimate, modified least-square estimate

## Abstract

The geometric process (GP) is a simple and direct approach to modeling of the successive inter-arrival time data set with a monotonic trend. In addition, it is a quite important alternative to the non-homogeneous Poisson process. In the present paper, the parameter estimation problem for GP is considered, when the distribution of the first occurrence time is Power Lindley with parameters α and λ. To overcome the parameter estimation problem for GP, the maximum likelihood, modified moments, modified L-moments and modified least-squares estimators are obtained for parameters *a*, α and λ. The mean, bias and mean squared error (MSE) values associated with these estimators are evaluated for small, moderate and large sample sizes by using Monte Carlo simulations. Furthermore, two illustrative examples using real data sets are presented in the paper.

## 1. Introduction

The Renewal process (RP) is a commonly used method for the statistical analysis of the successive inter-arrival times data set observed from a counting process. When the data set is non-trending, independently and identically distributed (iid), RP is a possible approach to modeling the data set. However, if the data follow a monotone trend, the data set can be modeled by a more possible approach than RP, such as the non-homogeneous Poisson process with a monotone intensity function, or a GP.

GP is first introduced as a direct approach to modeling of the inter-arrival times data with the monotone trend by Lam [1]. Actually, GP is a generalization of the RP with a ratio parameter. However, GP is a more flexible approach than RP for modeling of the successive inter-arrival time data with a trend. Because of this feature, GP has been successfully used as a model in many real-life problems from science, engineering, and health.

Before progressing further, let us recall the following definition of GP given in [2].

**Definition** **1.** *Let $X_{i}$ be the inter-arrival time the (i−1)th and ith events of a counting process $\left\{N(t),t\geq 0\right\}$ for i=1,2,⋯. Then, the stochastic process $\left\{X_{n},n=1,2,\cdots \right\}$ generated by $X_{i}$ random variables is said to be a geometric process (GP) with parameter a if there exists a real number a>0 such that*

(1) $$Y_{i}=a^{i-1}X_{i},\;\;\;\;i=1,2,\cdots \;$$

*are iid random variables with the distribution function F, where a is called the ratio parameter of the GP.*

Clearly, the parameter *a* arranges monotonic behavior of the GP. In Table 1, the monotonic behavior of the GP is given.

In the GP, the assumption on the distribution of $X_{1}$ has a special significance because of the fact that the distribution of $X_{1}$ and the other random variables $X_{2},\cdots ,X_{n}$ are from the same family of distributions with a different set of parameters. Namely, $X_{i}$s $\left(i=1,2,\cdots ,n\right)$ are distributed independently, but not identically. This is trivial from Definition 1. By considering this property, the expectation and variance of $\;X_{i}$s are immediately obtained as

(2) $$E\left(X_{i}\right)=\frac{\mu }{a^{i-1}}\;\;\;\;\;\;\;\;i=1,2,\cdots ,$$

(3) $$Var\left(X_{i}\right)=\frac{\sigma^{2}}{a^{2\left(i-1\right)}},\;\;\;\;\;\;\;\;i=1,2,\cdots ,$$

where μ and $\sigma^{2}$ are the expectation and variance of the random variable $X_{1,}$ respectively. Since the distribution of the random variable $X_{1}$ determines the distribution of the other variables, the selection of the distribution of $X_{1}$ based on the observed data is quite important to optimal statistical inference [3,4]. There are many studies in the literature on the solution to the parameter estimation problem of GP while selecting some special distributions for $X_{1}$. Chan et al. [5] investigated the parameter estimation problem of GP by assuming that the distribution of random variable $X_{1}$ was Gamma. Lam et al. [6] investigated the statistical inference problem for GP with Log-Normal distribution according to parametric and non-parametric methods. When the distribution of random variable $X_{1}$ was the inverse Gaussian, Rayleigh, two-parameter Rayleigh and Lindley, the problems of statistical inference for GP were investigated according to ML and modified moment method by [7,8,9,10], respectively.

The main objective of this study extensively investigates the solution of parameter estimation problem for GP when the distribution of the first arrival time is Power Lindley. In accordance with this objective, estimators for the parameters of GP with the Power Lindley distribution are obtained according to methods of maximum likelihood (ML), modified moment (MM), modified L-moment (MLM) and modified least-squares (MLS). The method of moments and the least-squares estimators of Power Lindley distribution are available in the literature [11]. The L-moments estimator of the Power Lindley distribution is obtained with this paper. In addition, the novelty of this paper is that the distribution of first inter-arrival time is assumed to be Power Lindley for GP and the ML and MLM estimators under this assumption are obtained.

The rest of this study is organized as follows: Section 2 includes the detailed infomation about the Power Lindley Distribution. In Section 3, the ML, the MLS, the MM and the MLM estimators of the parameters a, α and λ are obtained. Section 4 presents the results of performed Monte Carlo simulations for comparing the performances of the estimators obtained in Section 3. For illustrative purposes, two examples with real data sets are given in Section 5. Section 6 concludes the study.

## 2. An Overview to Power Lindley Distribution

The Power Lindley distribution was originally introduced by Githany et al. [11] as an important alternative for modeling of failure times. The distribution has a powerful modeling capability for positive data from different areas, such as reliability, lifetime testing, etc. In addition, for modeling the data sets with various shapes, many different extensions of the Power Lindley distribution have been attempted by researchers under different scenarios [12,13,14,15,16,17,18,19,20].

**Figure 1:**
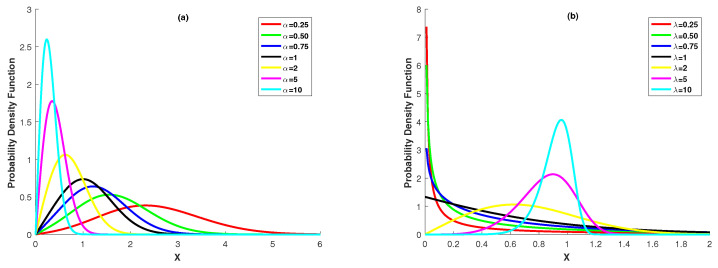
Pdf of the Power Lindley distribution (**a**): α=0.25,0.50,0.75,1,2,5,10 and λ=2; (**b**) α=2 and λ=0.25,0.50,0.75,1,2,5,10.

Let *X* be a Power Lindley distributed random variable with the parameters α and λ. From now on, a Power Lindley distributed random variable *X* will be indicated as $X∼PL\left(\alpha ,\lambda \right)$ for brevity. The probability density function (pdf) of the random variable *X* is

(4) $$f\left(x;\alpha ,\lambda \right)=\frac{\alpha \lambda^{2}}{1+\lambda }\left(1+x^{\alpha }\right)x^{\alpha -1}e^{-\lambda x^{\alpha }},\;\;\;\;\;x>0,\;\lambda >0,\;\alpha >0$$

and the corresponding cumulative distribution function (cdf) is

(5) $$F\left(x,\alpha ,\lambda \right)=1-\left(1+\frac{\lambda }{\lambda +1}x^{\alpha }\right)e^{-\lambda x^{\alpha }},\;\;\;\;\;x>0,$$

where α and λ are the positive and real-valued scale parameter and shape parameter of the distribution, respectively. Essentially, the Power Lindley distribution is a two-component mixture distribution with mixing ratio $\frac{\lambda }{1+\lambda }$ in which the first component is a Weibull distribution with parameters α and λ and the second component is a generalized Gamma distribution with parameter 2, α and λ. The Power Lindley distribution is a quite important alternative to standard distribution families for analyzing of lifetime data, since its distribution function, survival function and hazard function are expressed in explicit form. The Power Lindley distribution also has uni-mode and belongs to the exponential family. To clearly show the shape of the distribution, we present Figure 1. Figure 1a,b show the behavior of the pdf of Power Lindley distribution at the different values of the parameters α and λ.

Some basic measures of the Power Lindley distribution are tabulated in Table 2.

Advanced readers can refer to [11] for more information on the Power Lindley distribution.

### 2.1. Shannon and Rényi Entropy of the Power Lindley Distribution

The entropy is a measure of variation or uncertainty of a random variable. In this subsection, we investigate the Shannon and Rényi entropy, which are the two most popular entropies for Power Lindley distribution. The Shannon entropy (SE) of a random variable *X* with pdf *f* is defined as, see [21],

(6) $$H\left(X\right)=E\left\{-lnf(x)\right\}.$$

Then, by using the pdf (4), the SE of the Power Lindley distribution is found as

(7) $$\begin{array}{cc} H\left(X\right)=-E\left\{\ln f\left(x,\alpha ,\lambda \right)\right\} & =-\left\{\int_{0}^{\infty }\left[\ln\left(\frac{\alpha \lambda^{2}}{1+\lambda }\right)+\left(\alpha -1\right)\ln x-\lambda x^{\alpha }\right]f\left(x,\alpha ,\lambda \right)dx\right\}\;\\ & =-\left\{\ln\left(\frac{\alpha \lambda^{2}}{1+\lambda }\right)+\left(\alpha -1\right)\int_{0}^{\infty }\ln xf\left(x,\alpha ,\lambda \right)dx-\lambda \int_{0}^{\infty }x^{\alpha }f\left(x,\alpha ,\lambda \right)dx\right\}\;\\ & =-\left\{\ln\left(\frac{\alpha \lambda^{2}}{1+\lambda }\right)+\left(\alpha -1\right)E\left[\ln X\right]-\lambda E\left[X^{\alpha }\right]\right\}\;\\ & =-\left\{\ln\left(\frac{\alpha \lambda^{2}}{1+\lambda }\right)+\left(\alpha -1\right)E\left[\ln X\right]-\lambda E\left[X^{\alpha }\right]\right\}\;\\ & =\lambda \frac{\alpha \left[\alpha \left(\lambda +1\right)+\alpha \right]}{\alpha^{2}\left(\lambda +1\right)}-\left(\alpha -1\right)\frac{1-\left(1+\lambda \right)\left[\Psi \left(1\right)+\ln\lambda \right]}{\alpha \left(1+\lambda \right)}-\ln\left(\frac{\alpha \lambda^{2}}{1+\lambda }\right), \end{array}$$

where $\Psi \left(.\right)$ is the digamma function [22]. The Rényi entropy of a random variable with pdf *f* is defined as

(8) $$RE_{X}\left(\xi \right)=\frac{1}{1-\xi }\log\left\{\int_{-\infty }^{\infty }{\left[f\left(x\right)\right]}^{\xi }dx\right\}.$$

By using the pdf (4), Rényi entropy of the Power Lindley distribution is obtained as follows:

(9) $$\begin{array}{cc} RE_{X}\left(\xi \right) & =\frac{1}{1-\xi }\log\left\{\int_{-\infty }^{\infty }{\left[\frac{\alpha \lambda^{2}}{1+\lambda }\left(1+x^{\alpha }\right)x^{\alpha -1}e^{-\lambda x^{\alpha }}\right]}^{\xi }dx\right\}\\ & =\frac{1}{1-\xi }\log\left\{{\left(\frac{\alpha \lambda^{2}}{1+\lambda }\right)}^{\xi }\int_{-\infty }^{\infty }{\left(x^{\alpha -1}e^{-\lambda x^{\alpha }}\right)}^{\xi }{\left(1+x^{\alpha }\right)}^{\xi }dx\right\}. \end{array}$$

Applying the power expansion formula and gamma function to Equation (9), $RE_{X}\left(\xi \right)$ is obtained as

(10) REXξ=11−ξlogαλ21+λξ∑i=0ξξi∫−∞∞xα−1e−λxαξxξαdx=11−ξlogαλ21+λξ∑i=0ξξi(λξ)−2αξ+ξ−1αΓ2αξ−ξ+1αα.

## 3. Estimation of Parameters of GP with Power Lindley Distribution

### 3.1. Maximum Likelihood Estimation

Let $X_{1},X_{2},\cdots ,X_{n}$ be a random sample from a GP with ratio *a* and $X_{1}∼PL\left(\alpha ,\lambda \right)$. The likelihood function for $X_{i},i=1,2,\cdots ,n$ is

(11) $$L\left(a,\alpha ,\lambda \right)=a^{\frac{n\left(n-1\right)}{2}}\frac{\alpha^{n}\lambda^{2n}}{{\left(\lambda +1\right)}^{n}}\prod_{i=1}^{n}\left(1+{\left(a^{i-1}x_{i}\right)}^{\alpha }\right){\left(a^{i-1}x_{i}\right)}^{\alpha -1}e^{-\lambda {\left(a^{i-1}x_{i}\right)}^{\alpha }}.$$

From Equation (11), the corresponding log-likelihood function can be written as below:

(12) $$\begin{array}{cc} \ln L(a,\alpha ,\lambda ) & =\frac{n\left(n-1\right)}{2}\ln a+n\left[\ln\left(\alpha \right)+2\ln\lambda -\ln\left(\lambda +1\right)\right]+\;\\ & \;\;\;\;\sum_{i=1}^{n}\ln\left(1+{\left(a^{i-1}x_{i}\right)}^{\alpha }\right)+\left(\alpha -1\right)\sum_{j=1}^{n}\ln\left(a^{j-1}x_{j}\right)-\lambda \sum_{i=1}^{n}{\left(a^{i-1}x_{i}\right)}^{\alpha }. \end{array}$$

If the first derivatives of Equation (12) with respect to a, α and λ are taken, we have the following likelihood equations:

(13) $$\begin{array}{cc} \frac{\partial \ln L(a,\alpha ,\lambda )}{\partial a} & =\alpha \frac{n\left(n-1\right)}{2a}+\alpha \sum_{i=1}^{n}a^{i-2}\frac{{\left(a^{i-1}x_{i}\right)}^{\alpha -1}}{{\left(a^{i-1}x_{i}\right)}^{\alpha }+1}x_{i}\left(i-1\right)\\ & \;\;\;\;\;-\lambda \sum_{i=1}^{n}\alpha {\left(a^{i-1}x_{i}\right)}^{\alpha -1}x_{i}\left(i-1\right)a^{i-2}\left.=0,\right. \end{array}$$

(14) $$\begin{array}{cc} \frac{\partial \ln L(a,\alpha ,\lambda )}{\partial \alpha } & =\frac{n}{\alpha }+\sum_{i=1}^{n}\left(\ln a^{i-1}x_{i}\right)\frac{{\left(a^{i-1}x_{i}\right)}^{\alpha }}{{\left(a^{i-1}x_{i}\right)}^{\alpha }+1}+\sum_{i=1}^{n}\ln\left(a^{i-1}x_{i}\right)\\ & \;\;\;\;\;\;-\lambda \sum_{i=1}^{n}\left(\ln\frac{1}{a}a^{i}x_{i}\right){\left(\frac{1}{a}a^{i}x_{i}\right)}^{\alpha }\left.=0\right. \end{array}$$

and

(15) $$\frac{\partial \ln L(a,\alpha ,\lambda )}{\partial \lambda }=\frac{2n}{\lambda }+\frac{n}{\lambda +1}-\sum_{i=1}^{n}{\left(a^{i-1}x_{i}\right)}^{\alpha }=0.$$

From the solution of likelihood Equations (13)–(15), the parameter λ is obtained as

(16) $$\lambda \left(\hat{a},\hat{\alpha }\right)=\frac{1-{\bar{X}}_{a\alpha }+\sqrt{6{\bar{X}}_{a\alpha }+{\bar{X}}_{a\alpha }^{2}+1}}{2{\bar{X}}_{a\alpha }},$$

where ${\bar{X}}_{a\alpha }=\frac{\sum_{i=1}^{n}{\left({\hat{a}}^{i-1}x_{i}\right)}^{\hat{\alpha }}}{n}$. However, analytical expressions for the ML estimators of the parameters *a* and α cannot be obtained from likelihood Equations (13)–(15). In order to estimate these parameters, Equations (13)–(15) must be simultaneously solved by using a numerical method such as Newton’s method.

Let $\theta =\left[\begin{array}{c} a\\ \alpha \\ \lambda \end{array}\right]$ be the parameter vector and $\nabla \left(\theta \right)$ corresponding gradient vector for this parameter vector, i.e.,

(17) $$\nabla \left(\theta \right)=\left[\begin{array}{c} \frac{\partial \ln L(a,\lambda )}{\partial a}\\ \frac{\partial \ln L(a,\lambda )}{\partial \alpha }\\ \frac{\partial \ln L(a,\lambda )}{\partial \lambda } \end{array}\right].$$

Under these notations, Newton’s method is given as

(18) $$\theta_{m+1}=\theta_{m}-H^{-1}\left(\theta_{m}\right)\nabla \left(\theta_{m}\right),$$

where *m* is the iteration number and $H^{-1}\left(\theta \right)$ is the inverse of the Hessian matrix $H\left(\theta \right)$, $H\left(\theta \right)\in R^{3\times 3}$. The elements of the matrix $H\left(\theta \right)$ are of the second derivatives of the log-likelihood function given in Equation (11) with respect to parameters a, α and λ. Let $h_{ij}$ be the $\left(i,j\right)th$ $\left(i,j=1,2,3\right)$ element of the matrix $H\left(\theta \right)$. The $h_{ij}$s are obtained as below:

$$\begin{array}{c} h_{11}=\frac{\partial^{2}\ln L}{\partial a^{2}}=-\alpha \frac{n\left(n-1\right)}{2a^{2}}+\alpha \sum_{i=1}^{n}\left(-\frac{1}{a^{2}}\frac{{\left(a^{i-1}x_{i}\right)}^{\alpha }}{{\left({\left(a^{i-1}x_{i}\right)}^{\alpha }+1\right)}^{2}}\left(i-1\right)\left(\alpha -i\alpha +{\left(a^{i-1}x_{i}\right)}^{\alpha }+1\right)\right)\\ -\lambda \sum_{i=1}^{n}\left(-\frac{1}{a^{2}}\alpha {\left(a^{i-1}x_{i}\right)}^{\alpha }\left(i-1\right)\left(\alpha -i\alpha +1\right)\right), \end{array}$$

$$\begin{array}{cc} h_{12} & =\frac{\partial^{2}\ln L}{\partial a\partial \alpha }=\frac{n\left(n-1\right)}{2a}+\sum_{i=1}^{n}\frac{{\left(a^{i-1}x_{i}\right)}^{\alpha }}{a+a{\left(a^{i-1}x_{i}\right)}^{\alpha }}\left(i-1\right)\\ & +\alpha \sum_{i=1}^{n}\frac{1}{a}\left(\ln a^{i-1}x_{i}\right)\frac{{\left(a^{i-1}x_{i}\right)}^{\alpha }}{{\left({\left(a^{i-1}x_{i}\right)}^{\alpha }+1\right)}^{2}}\left(i-1\right)\\ & -\lambda \sum_{i=1}^{n}a^{i-2}{\left(a^{i-1}x_{i}\right)}^{\alpha -1}\left(\alpha \ln a^{i-1}x_{i}+1\right)x_{i}\left(i-1\right), \end{array}$$

$$h_{13}=\frac{\partial^{2}\ln L}{\partial a\partial \lambda }=-\alpha \sum_{i=1}^{n}{\left(a^{i-1}x_{i}\right)}^{\alpha -1}x_{i}\left(i-1\right)a^{i-2},$$

$$\begin{array}{c} h_{21}=\frac{\partial^{2}\ln L}{\partial \alpha \partial a}=\frac{n\left(n-1\right)}{2a}+\frac{1}{a}\sum_{i=1}^{n}\frac{{\left(a^{i-1}x_{i}\right)}^{\alpha }}{{\left({\left(a^{i-1}x_{i}\right)}^{\alpha }+1\right)}^{2}}\left(i-1\right)\left(\alpha \ln a^{i-1}x_{i}+{\left(a^{i-1}x_{i}\right)}^{\alpha }+1\right)\\ -\lambda \sum_{i=1}^{n}\frac{1}{a}{\left(a^{i-1}x_{i}\right)}^{\alpha }\left(\alpha \ln a^{i-1}x_{i}+1\right)\left(i-1\right), \end{array}$$

$$h_{22}=\frac{\partial^{2}\ln L}{\partial \alpha^{2}}=-\frac{n}{\alpha^{2}}+\sum_{i=1}^{n}{\left(\ln a^{i-1}x_{i}\right)}^{2}\frac{{\left(a^{i-1}x_{i}\right)}^{\alpha }}{{\left({\left(a^{i-1}x_{i}\right)}^{\alpha }+1\right)}^{2}}-\lambda \sum_{i=1}^{n}{\left(\ln a^{i-1}x_{i}\right)}^{2}{\left(a^{i-1}x_{i}\right)}^{\alpha },$$

$$h_{23}=\frac{\partial^{2}\ln L}{\partial \alpha \partial \lambda }=-\sum_{i=1}^{n}\left(\ln a^{i-1}x_{i}\right){\left(a^{i-1}x_{i}\right)}^{\alpha },$$

$$h_{31}=\frac{\partial^{2}\ln L}{\partial \lambda \partial a}=-\alpha \sum_{i=1}^{n}{\left(a^{i-1}x_{i}\right)}^{\alpha -1}x_{i}\left(i-1\right)a^{i-2},$$

$$h_{32}=\frac{\partial^{2}\ln L}{\partial \lambda \partial a}=-\sum_{i=1}^{n}\left(\ln a^{i-1}x_{i}\right){\left(a^{i-1}x_{i}\right)}^{\alpha },$$

$$h_{33}=\frac{\partial^{2}\ln L}{\partial \lambda^{2}}=-\frac{2n}{\lambda^{2}}-\frac{n}{{\left(\lambda +1\right)}^{2}}.$$

Hence, $H^{-1}\left(\theta \right)$ is obtained as

(19) $$H^{-1}\left(\theta \right)=\frac{1}{Det\left(H\left(\theta \right)\right)}\left[\begin{array}{ccc} h_{22}h_{33}-h_{23}h_{32} & -h_{12}h_{33}-h_{13}h_{32} & h_{12}h_{23}-h_{13}h_{22}\\ -h_{21}h_{33}-h_{31}h_{23} & h_{11}h_{33}-h_{13}h_{31} & -h_{11}h_{23}-h_{21}h_{13}\\ h_{21}h_{32}-h_{22}h_{31} & -h_{11}h_{32}-h_{12}h_{31} & h_{11}h_{22}-h_{12}h_{21} \end{array}\right],$$

where $Det\left(H\left(\theta \right)\right)$ is the determinant of the matrice $H\left(\theta \right)$ and it is calculated by

(20) $$Det\left(H\left(\theta \right)\right)=h_{11}h_{22}h_{33}-h_{11}h_{23}h_{32}-h_{12}h_{21}h_{33}+h_{12}h_{31}h_{23}+h_{21}h_{13}h_{32}-h_{13}h_{22}h_{31}.$$

Thus, by starting with being given an initial estimation $\theta_{0}$, the parameter vector θ can be estimated with an iterative method given by (18). Hence, the ML estimates of the parameters a, α and λ, say ${\hat{a}}_{ML}$, ${\hat{\alpha }}_{ML}$ and ${\hat{\lambda }}_{ML},$ respectively, are obtained as respective elements of the $\theta_{m}$. The joint distribution of ${\hat{a}}_{ML}$, ${\hat{a}}_{ML}$ and ${\hat{a}}_{ML}$ estimators is asymptotically normal with mean vector $\left(a,\alpha ,\lambda \right)$ and covariance matrix $I^{-1}\left(a,\alpha ,\lambda \right),$ (see, [23]), where *I* is the Fisher information matrix, i.e.,

(21) $$I=-\left[\begin{array}{ccc} E\left(\frac{\partial \ln L(a,\alpha ,\lambda )}{\partial a^{2}}\right) & E\left(\frac{\partial \ln L(a,\alpha ,\lambda )}{\partial a\partial \alpha }\right) & E\left(\frac{\partial \ln L(a,\alpha ,\lambda )}{\partial a\partial \lambda }\right)\\ E\left(\frac{\partial \ln L(a,\lambda )}{\partial \alpha \partial a}\right) & E\left(\frac{\partial \ln L(a,\alpha ,\lambda )}{\partial \alpha^{2}}\right) & E\left(\frac{\partial \ln L(a,\alpha ,\lambda )}{\partial a\partial \lambda }\right)\\ E\left(\frac{\partial \ln L(a,\lambda )}{\partial \lambda \partial a}\right) & E\left(\frac{\partial \ln L(a,\alpha ,\lambda )}{\partial \lambda \partial \alpha }\right) & E\left(\frac{\partial \ln L(a,\alpha ,\lambda )}{\partial \lambda^{2}}\right) \end{array}\right].$$

The elements of the Fisher information matrix *I* given by (21) are immediately written from elements of the Hessian matrix $H\left(\theta \right)$. However, an explicit form of the Fisher information matrix *I* cannot be derived. Fortunately, as an estimator, the observed information matrix can be used instead of matrix *I*. Note that the observed information matrix of the estimators is the negative value of the matrix $H\left(\theta \right)$ obtained at the last iteration.

### 3.2. Modified Methods

Since GP is a monotonic stochastic process, some divergence problems may arise in the estimation stage of the ratio parameter *a*. To overcome this problem, estimating the ratio parameter *a* by nonparametrically is a widely used method in statistical inference for GP [2,24]. A nonparametric estimator for the ratio parameter *a* is given by, see [6,25],

(22) $${\hat{a}}_{NP}=\exp\left(\frac{6}{\left(n-1\right)n\left(n+1\right)}\sum_{i=1}^{n}\left(n-2i+1\right)\ln X_{i}\right).$$

The estimator ${\hat{a}}_{NP}$ is an unbiased estimator and follows the asymptotic normal distribution, see [25]. When the ${\hat{a}}_{NP}$ given by (22) is substituted into Equation (1), it can be immediately written

(23) $${\hat{Y}}_{i}={\hat{a}}_{NP}^{i-1}X_{i},\;\;\;\;\;i=1,2,\cdots ,n.$$

Thus, the parameters α and λ can be estimated with a selected estimation method by using the estimators ${\hat{Y}}_{i}$ $\left(i=1,2,\cdots ,n\right)$. This estimation rule is called modified method (see [6]).

#### 3.2.1. MM Estimation

Let $X_{1},X_{2},\cdots ,X_{n}$ be a random sample from a GP with ratio *a* and $X_{1}∼PL\left(\alpha ,\lambda \right)$. In addition, we assume that the ratio parameter *a* is nonparametrically estimated by (22). For the sample $X_{1},X_{2},\cdots ,X_{n}$, first and second sample moments, $m_{1}$ and $m_{2},$ are calculated by

(24) $$m_{1}=\frac{1}{n}\sum_{i=1}^{n}{\hat{a}}_{NP}^{i-1}X_{i}$$

and

(25) $$m_{2}=\frac{1}{n}\sum_{i=1}^{n}{\hat{a}}_{NP}^{2\left(i-1\right)}X_{i}^{2},$$

respectively. On the other hand, from Table 2, first and second population moments of the distribution PL(α,λ), say $\mu_{1}$ and $\mu_{2}$, can be easily written as

(26) $$\mu_{1}=\frac{\Gamma \left(\frac{1}{\alpha }\right)\left[\alpha \left(\lambda +1\right)+1\right]}{\alpha^{2}\lambda^{1/\alpha }\left(\lambda +1\right)}$$

and

(27) $$\mu_{2}=\frac{2\Gamma \left(\frac{2}{\alpha }\right)\left[\alpha \left(\lambda +1\right)+2\right]}{\alpha^{2}\lambda^{2/\alpha }\left(\lambda +1\right)},$$

respectively. Thus, the MM estimators of the parameters α and λ, ${\hat{\alpha }}_{MM}$ and ${\hat{\lambda }}_{MM},$ respectively, can be obtained from the solution of the following nonlinear equation system:

(28) $$\begin{array}{cc} \frac{\Gamma \left(\frac{1}{\alpha }\right)\left[\alpha \left(\lambda +1\right)+1\right]}{\alpha^{2}\lambda^{1/\alpha }\left(\lambda +1\right)}-m_{1} & =0\\ \frac{2\Gamma \left(\frac{2}{\alpha }\right)\left[\alpha \left(\lambda +1\right)+2\right]}{\alpha^{2}\lambda^{2/\alpha }\left(\lambda +1\right)}-m_{2} & =0 \end{array}$$

#### 3.2.2. MLM Estimation

In this subsection, the MLM estimators ${\hat{\alpha }}_{MLM}$ and ${\hat{\lambda }}_{MLM}$ are obtained for the parameters α and λ, respectively, when the ratio parameter *a* is estimated by (22). L-moments’ estimators have been proposed as a method based on the linear combination of the order statistics by Hosking [26]. Due to their useful and robust structure, the L-moments estimators have been intensively studied and, in order to estimate the unknown parameters of many probability distributions, L-moment estimators have been obtained. For more information about the L-moments, we refer the readers to [26].

As in the method of moments, to obtain the L-moment estimators, population L-moments are equated to sample L-moments. In our problem, the first two samples and population L-moments are necessary for obtaining the estimators ${\hat{\alpha }}_{MLM}$ and ${\hat{\lambda }}_{MLM}$. Under the transform ${\hat{Y}}_{i}={\hat{a}}^{i-1}X_{i}$, $\left(i=1,\cdots ,n\right),$ first and second sample L-moments are calculated as follows, see [26]:

(29) $$l_{1}=\frac{1}{n}\sum_{i=1}^{n}{\hat{Y}}_{\left(i\right)},$$

(30) $$l_{2}=\frac{2}{n\left(n-1\right)}\sum_{i=1}^{n}\left(i-1\right){\hat{Y}}_{\left(i\right)}-l_{1},$$

where ${\hat{Y}}_{\left(i\right)}$, $\left(i=1,2,\cdots ,n\right)$ represents the ordered observations. On the other hand, using the notations in [26], first and second population L-moments of $PL\left(\lambda ,\alpha \right)$ are

(31) $$\begin{array}{cc} L_{1} & =\frac{\lambda }{\lambda +1}\frac{\Gamma \left(\frac{1}{\alpha }+1\right)}{\lambda^{\frac{1}{\alpha }}}+\frac{1}{\lambda +1}\frac{\Gamma \left(\frac{1}{\alpha }+2\right)}{\lambda^{\frac{1}{\alpha }}}\\ & =\frac{\Gamma \left(\frac{1}{\alpha }\right)\left(\alpha +\alpha \lambda +1\right)}{\alpha^{2}\lambda^{\frac{1}{\alpha }}\left(\lambda +1\right)} \end{array}$$

and

(32) $$\begin{array}{cc} L_{2} & =\left\{\frac{\Gamma \left(\frac{1}{\alpha }\right)\left(\alpha \left(\lambda +1\right)+1\right)}{\alpha^{2}\lambda^{\frac{1}{\alpha }}\left(\lambda +1\right)}-\frac{2\alpha \lambda^{2}}{1+\lambda }\left(\frac{\Gamma \left(\frac{\alpha +1}{\alpha }\right)}{\alpha {\left(2\lambda \right)}^{\frac{\alpha +1}{\alpha }}}+\frac{\Gamma \left(\frac{2\alpha +1}{\alpha }\right)}{\alpha {\left(2\lambda \right)}^{\frac{2\alpha +1}{\alpha }}}\right)-\right.\\ & \left.\frac{2\alpha \lambda^{3}}{{\left(1+\lambda \right)}^{2}}\left(\frac{\Gamma \left(\frac{2\alpha +1}{\alpha }\right)}{\alpha {\left(2\lambda \right)}^{\frac{2\alpha +1}{\alpha }}}+\frac{\Gamma \left(\frac{3\alpha +1}{\alpha }\right)}{\alpha {\left(2\lambda \right)}^{\frac{3\alpha +1}{\alpha }}}\right)\right\}. \end{array}$$

See Appendix A for the derivation of population L-moments.

Thus, the estimators ${\hat{\alpha }}_{MLM}$ and ${\hat{\lambda }}_{MLM}$ are obtained from the numerical solution of the following nonlinear system:

(33) $$\begin{array}{c} \frac{\Gamma \left(\frac{1}{\alpha }\right)\left(\alpha +\alpha \lambda +1\right)}{\alpha^{2}\lambda^{\frac{1}{\alpha }}\left(\lambda +1\right)}-l_{1}=0,\\ \left\{\frac{\Gamma \left(\frac{1}{\alpha }\right)\left(\alpha \left(\lambda +1\right)+1\right)}{\alpha^{2}\lambda^{\frac{1}{\alpha }}\left(\lambda +1\right)}-\frac{2\alpha \lambda^{2}}{1+\lambda }\left(\frac{\Gamma \left(\frac{\alpha +1}{\alpha }\right)}{\alpha {\left(2\lambda \right)}^{\frac{\alpha +1}{\alpha }}}+\frac{\Gamma \left(\frac{2\alpha +1}{\alpha }\right)}{\alpha {\left(2\lambda \right)}^{\frac{2\alpha +1}{\alpha }}}\right)-\right. \end{array}$$

(34) $$\begin{array}{c} \left.\frac{2\alpha \lambda^{3}}{{\left(1+\lambda \right)}^{2}}\left(\frac{\Gamma \left(\frac{2\alpha +1}{\alpha }\right)}{\alpha {\left(2\lambda \right)}^{\frac{2\alpha +1}{\alpha }}}+\frac{\Gamma \left(\frac{3\alpha +1}{\alpha }\right)}{\alpha {\left(2\lambda \right)}^{\frac{3\alpha +1}{\alpha }}}\right)\right\}-l_{2}=0. \end{array}$$

#### 3.2.3. MLS Estimation

The least-squares estimator (LSE) is a regression-based method proposed by Swain et al. [27]. Essentially, the method is a nonlinear curve fitting for the cdf of a random variable by using the empirical distribution function. In the least-squares method, the estimator(s) is determined such that the squared difference between the empirical distribution function and the fitted curve is minimum. Let $Z_{1},\cdots ,Z_{n}$ be a random sample from a distribution function $F_{Z}\left(.\right)$. In addition, the order statistics of the random sample $Z_{1},\cdots ,Z_{n}$ are represented by $Z_{\left(1\right)},\cdots ,Z_{\left(n\right)}$. In this situation, the LSE of the parameters are obtained minimizing

(35) $$\sum_{j=1}^{n}{\left(F_{Z}\left(Z_{\left(i\right)}\right)-E\left(F_{Z}(Z_{\left(i\right)}\right)\right)}^{2}$$

with respect to the parameters of $F_{Z}\left(.\right)$ [27], where expectation $E\left(F_{Z}(Z_{\left(i\right)}\right)$ is calculated by

(36) $$E\left(F_{Z}(Z_{\left(i\right)}\right)=\frac{i}{n+1},\;\;\;\;i=1,2,\cdots ,n.$$

Therefore, in our problem, the MLS estimators of parameters α and λ, say ${\hat{\alpha }}_{MLS}$ and ${\hat{\lambda }}_{MLS}$, respectively, are obtained by minimizing

(37) $$\sum_{j=1}^{n}{\left(1-\left(1+\frac{\lambda }{\lambda +1}Y_{\left(j\right)}^{\alpha }\right)e^{-\lambda Y_{\left(j\right)}^{\alpha }}-\frac{j}{n+1}\right)}^{2}$$

with respect to α and λ.

## 4. Simulation Study

In order to evaluate the estimation performances of the ML, the MLS, the MM and the MLM estimators obtained in the previous section, some Monte Carlo simulation experiments are presented in this section. Throughout the simulation experiments, the scale parameter λ is assumed to be 0.5, without loss of generality and also the parameter α set as 0.5,1,1.5 and 2. For different sample size n=30,50,100,150 and ratio parameter a=0.90,0.95,1.05,1.10, values of means, biases and MSEs have been calculated for estimating the ML, the MLS, the MM and the MLM. The obtained results based on 10,000 replications are displayed in Table 3, Table 4, Table 5 and Table 6.

When the results of the simulation experiments given in Table 3, Table 4, Table 5 and Table 6 are analyzed, it is seen that the ML estimators have smaller MSE values than the other estimators in all cases. Therefore, the ML estimators outperform the modified estimators with smaller bias and MSE. In addition, when the sample size *n* increases, the values of the bias and MSE decrease for all estimators. Based on this result, it can be said that all estimators obtained in the previous section are asymptotically unbiased and consistent.

## 5. Illustrative Examples

Practical applications of the parameter estimation with developed procedures in Section 3 are illustrated in this section with two data sets called Aircraft data set and Coal mining disaster data set.

In the examples, we use two criteria, the mean-squared error (MSE*) [28] for the fitted values and the maximum percentage error (MPE), which are defined in [25], for comparing the stochastic processes GP and RP. The MSE* and MPE are described as follows:

- MSE* = $\left(1/n\right)\sum_{k=1}^{n}{\left(X_{k}-{\hat{X}}_{k}\right)}^{2},$
- MPE = $\max_{1\leq k\leq n}\left\{\left|Sk-{\hat{S}}_{k}\right|/S_{k}\right\},$

where ${\hat{X}}_{k}$ is calculated by

(38) $${\hat{X}}_{k}=\left\{\begin{array}{cc} {\hat{\mu }}_{ML}{\hat{a}}_{ML}^{1-k} & \mathrm{GP}\;\mathrm{with}\;\mathrm{the}\;\mathrm{ML}\;\mathrm{estimators},\;\;\;\\ {\hat{\mu }}_{MLS}{\hat{a}}_{MLS}^{1-k} & \mathrm{GP}\;\mathrm{with}\;\mathrm{the}\;\mathrm{MLS}\;\mathrm{estimators},\;\\ {\hat{\mu }}_{MM}{\hat{a}}_{MM}^{1-k} & \mathrm{GP}\;\mathrm{with}\;\mathrm{the}\;\mathrm{MM}\;\mathrm{estimators},\;\;\\ {\hat{\mu }}_{MLM}{\hat{a}}_{MLM}^{1-k} & \mathrm{GP}\;\mathrm{with}\;\mathrm{the}\;\mathrm{MLM}\;\mathrm{estimators},\\ {\hat{\mu }}_{ML} & \mathrm{RP}\;\mathrm{with}\;\mathrm{the}\;\mathrm{MLestimators},\;\;\;\; \end{array}\right.$$

and $S_{k}=X_{1}+X_{2}+\cdots +X_{k},k=1,2,\cdots ,n$ and ${\hat{S}}_{k}=\sum_{j=1}^{k}{\hat{X}}_{j}$. Then, in order to compare the relative performances of the RP and four GPs with the ML, the MLS, the MM and the MLM estimators, the plot of $S_{k}$ and ${\hat{S}}_{k}$ against k,k=1,2,⋯,n can be used.

**Example** **1.** *Aircraft data.*

The aircraft dataset consists of 30 observations that deal with the air-conditioning system failure times of a Boeing 720 aircraft (aircraft number 7912). The aircraft dataset was originally studied by Proschan [29]. The successive failure times in aircraft dataset are 23, 261, 87, 7, 120, 14, 62, 47, 225, 71, 246, 21, 42, 20, 5, 12, 120, 11, 3, 14, 71, 11, 14, 11, 16, 90, 1, 16, 52, 95.

**Figure 2:**
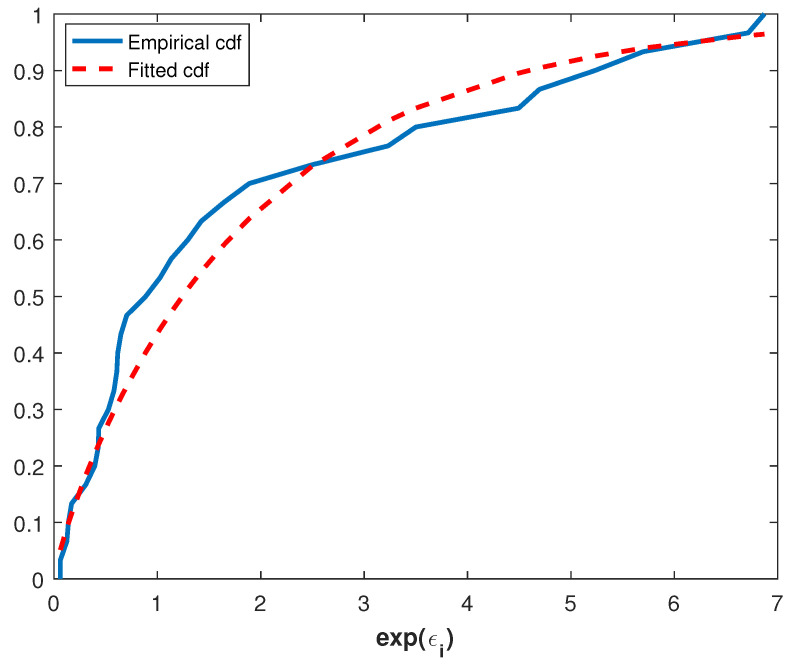
Empirical and fitted cdf of the $\exp({\hat{\varepsilon }}_{i})$.

We first investigate the underlying distribution of the set of data. To test whether the underlying distribution of the data $\left\{X_{1},\cdots ,X_{n}\right\}$ is the Power Lindley, the following procedures can be used. From Definition 1, we know that the $Y_{i}=a^{i-1}X_{i}$ and the $Y_{i}$s follow the Power Lindley. We can write immediately as $\ln Y_{i}=\left(i-1\right)\ln a+\ln X_{i}$ by taking the logarithm of $Y_{i}$. Note that $\ln Y_{i}$s follow the Log-Power Lindley distribution. Therefore, a linear regression model

(39) $$\ln X_{i}=\mu -\left(i-1\right)\ln a+\varepsilon_{i}$$

can be defined, where $\mu =E\left(\ln Y_{i}\right)$ and $\exp\left(\varepsilon_{i}\right)∼PL\left(\theta ,\beta \right)$. If the exponential errors are Power Lindley distributed, then the underlying distribution of the set of data is Power Lindley. The error term $\varepsilon_{i}$ in Equation (39) can be estimated by

(40) $${\hat{\varepsilon }}_{i}=\ln X_{i}-\hat{\mu }-\left(i-1\right)\ln{\hat{a}}_{NL},$$

where $\hat{\mu }=\frac{n\left(n-1\right)}{2}\ln{\hat{a}}_{NL}+\sum_{i=1}^{n}\ln X_{i}$. Thus, consistency of the exponentiated errors to Power Lindley distribution can be tested by using a goodness of fit test such as Kolmogorov–Smirnov (K-S). The parameter estimates of the exponentiated errors are θ=0.9717 and β=0.7985 and also the value of the K-S test is 0.1225 and the corresponding *p*-value is 0.7134. Therefore, it can be said that the underlying distribution of this data set is Power Lindley. This can also be seen from Figure 2, which illustrates the plots of the empirical and the fitted cdf.

When a GP with the Power Lindley distribution is used for modeling of this dataset, the ML, the MLS, the MM and the MLM estimates of the parameters a, α and λ and also MSE* and MPE values are tabulated in Table 3.

As it can be seen from Table 7, the ML estimates have the smallest MSE * values consistent with the simulation experiments. The estimator having the smallest MPE is the MM, but estimators of the ML and the MLM are very close to the MM.

Now, we check the model optimality. In order to select an optimal model to a data set, Akaike information criterion (AIC) and maximized log-likelihood (L) value are commonly used methods. For deciding an optimal model among the Power Lindley and its alternatives (Log-Normal, Gamma and inverse Gaussian) for this data set, we compute the -L and AIC values. The -L and AIC values for the models are given in Table 8.

**Figure 3:**
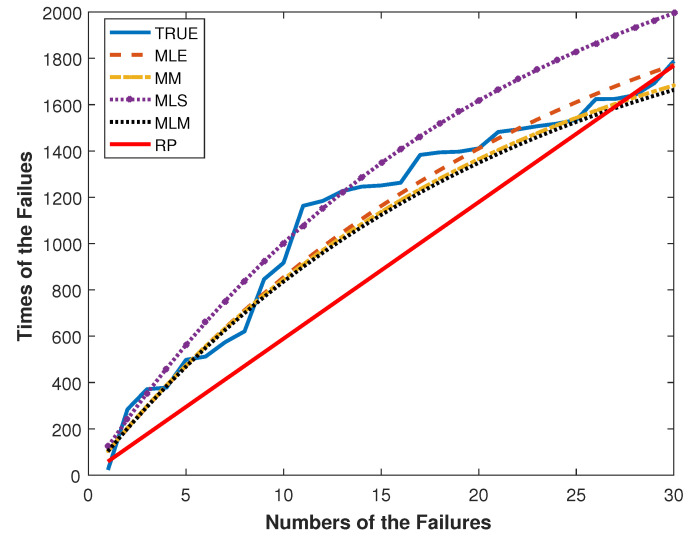
The plots of $S_{k}$ and ${\hat{S}}_{k}$ against the number of failures for the aircraft data.

The results given in Table 8 show that the Power Lindley distribution is an optimal model for the aircraft dataset with smaller AIC and -L values. In Figure 3, the failure times for the aircraft data and their fitted times are plotted.

The modeling performances of the RP and GP with the ML, the MLS, the MM and the MLM estimators can be easily compared by Figure 3. By Figure 3, the GP with the ML, the MLS, the MM and the MLM estimators outperform the RP. This result is compatible with Table 7.

**Example** **2.** *Coal mining disaster data.*

This example is from [30] on the intervals in days between successive disasters in Great Britain from 1851 to 1962. The coal mining disaster data set, which includes 190 successive intervals, was used as an illustrative example for GP by [7,10,24].

**Figure 4:**
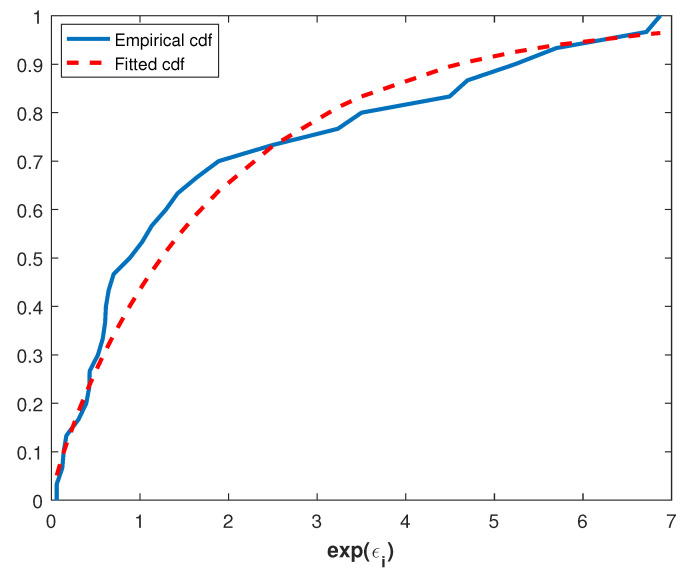
Q-Q plot of the coal mining disaster data.

For this data set, the value of K-S test is 0.0396 and the corresponding *p*-value is 0.9148. Thus, the Power Lindley distribution is an appropriate model for the coal mining disaster data. This is also supported by the following Q-Q plot, Figure 4, which is constructed by plotting the ordered exponential errors $\exp\left({\hat{\varepsilon }}_{i}\right)$ against the quantiles of the PL(0.9552,0.7930) because the data points fall approximately on the straight line in Figure 4.

For the coal mining disaster data set, the estimates of the parameters *a*, λ and α are given in Table 9.

Moreover, calculated AIC and L values for the coal mining disaster data set with the different models are tabulated in Table 10.

**Figure 5:**
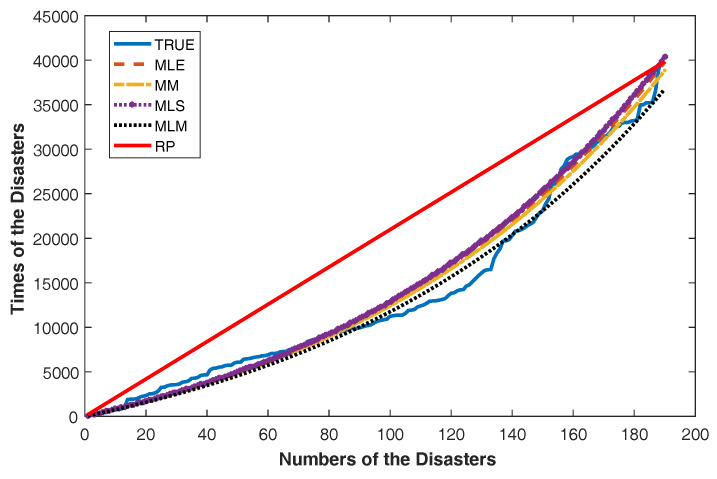
The plots $S_{k}$ and ${\hat{S}}_{k}$ against the number of failures for the coal mining disaster data.

We can say that the Power Lindley distribution is an optimal model for this data set since it has minimum AIC and -L values. Under the assumption that the underlying distribution of the data is Power Lindley, we present Figure 5 for comparing the modeling performance of the RP and the GP with four estimators obtained in the previous section. Figure 5 plots $S_{k}$ and ${\hat{S}}_{k}$ versus the number of disasters k, k=1,2,⋯,n.

As can be seen in Figure 5, the GP with the ML, the MLS, the MM and the MLM estimators more fairly follow real values than the RP. It can also be seen from Table 9 that the MSE* and MPE values of GP models are much smaller than RP. Thus, according to Figure 5 and Table 9, it is concluded that the GP provides the better data fit than RP.

## 6. Conclusions

In this paper, we have discussed the parameter estimation problem for GP by assuming that distribution of the first inter-arrival time is Power Lindley with parameters α and λ. In the paper, the parameter estimation problem has been solved from two points of view as the parametric (ML) and nonparametric (MLS, MM and MLM). Parametric estimators, ML, of the parameters A, ALF and LAM are also asymptotically normally distributed. However, more work should be done in order to say something about the asymptotic properties of nonparametric estimators MLS, MM and MLM. In addition, this is usually not an easy task because an analytical form of these estimators cannot be written.

Numerical study results have shown that the ML estimators outperform the MLS, MLM and MLS estimators with smaller bias and MSE measures. In addition, it has been observed that both bias and MSE values of all estimators decrease when the sample size increases. Hence, in light of numerical studies, it can be concluded that all of the estimators are asymptotically unbiased and consistent.

In the illustrative examples presented to demonstrate the data modeling performance of GP with the obtained estimators, the GP with Power Lindley distribution gives a better data fit than the RP in both examples. In addition, according to AIC and -L values, it can be said that modeling both the aircraft dataset and the coal mining disaster dataset using a GP with Power Lindley distribution is more appropriate than a GP with Gamma, log-normal or inverse Gaussian distribution. Therefore, it can be said that a GP with Power Lindley distribution is a quite important alternative to a GP with famous distributions such as Gamma, Log-normal or inverse Gaussian in modeling the successive inter-arrival times.

## Figures and Tables

**Table 1:** Behavior of GP according to values of ratio parameter *a*.

Value of Parameter *a*	Behavior of GP
*a* > 1	Stochastically decreasing process
*a* < 1	Stochastically increasing process
*a* = 1	Stable (GP is an RP)

**Table 2:** Some basic measures of the Power Lindley distributions.

Characteristic	Value
Expectation (μ)	$\frac{\Gamma \left(\frac{1}{\alpha }\right)\left[\alpha \left(\lambda +1\right)+1\right]}{\alpha^{2}\lambda^{1/\alpha }\left(\lambda +1\right)}$
*k*th moment ($\mu_{k}$)	$\frac{k\Gamma \left(\frac{k}{\alpha }\right)\left[\alpha \left(\lambda +1\right)+k\right]}{\alpha^{2}\lambda^{k/\alpha }\left(\lambda +1\right)}$
Variance ($\sigma^{2}$)	$\frac{2\Gamma \left(\frac{2}{\alpha }\right)\left[\alpha \left(\lambda +1\right)+2\right]\alpha^{2}\left(\lambda +1\right)-{\left[\Gamma \left(\frac{1}{\alpha }\right)\right]}^{2}{\left[\alpha \left(\lambda +1\right)+1\right]}^{2}}{\alpha^{4}\lambda^{2/\alpha }{\left(\lambda +1\right)}^{2}}$
Kurtosis	$\frac{\mu_{4}-4\mu_{3}\mu +6\mu_{2}\mu^{2}-3\mu^{4}}{\sigma^{4}}$
Skewness	$\frac{\mu_{3}-3\mu_{2}\mu +2\mu^{3}}{\sigma^{3}}$

**Table 3:** The simulated Means, Biases and *n* × MSE values for the MLE, MLS, MM and MLM estimators of the parameters *a*, α and λ, when α=0.5.

				$\hat{a}$				$\hat{\lambda }$				$\hat{\alpha }$	
*a*	*n*	Method	Mean	Bias	*n* × MSE		Mean	Bias	*n* × MSE		Mean	Bias	*n* × MSE
0.90	30	MLE	0.9002	0.0002	0.5273		0.5050	0.0050	12.8420		0.5283	0.0283	2.8920
		MLS	0.9006	0.0006	0.8838		0.5461	0.0461	20.1892		0.4977	−0.0023	3.3405
		MM	0.9006	0.0006	0.8838		0.4657	−0.0343	20.3422		0.5639	0.0639	6.6133
		MLM	0.9006	0.0006	0.8838		0.6382	0.1382	39.2676		0.4634	−0.0366	3.7622
	50	MLE	0.9002	0.0002	0.1227		0.5048	0.0048	7.4417		0.5172	0.0172	1.4622
		MLS	0.8994	−0.0006	0.2186		0.5342	0.0342	12.4524		0.5018	0.0018	1.9141
		MM	0.8994	−0.0006	0.2186		0.4877	−0.0123	15.3639		0.5397	0.0397	3.8289
		MLM	0.8994	−0.0006	0.2186		0.6302	0.1302	26.5101		0.4585	−0.0415	2.6689
	100	MLE	0.9002	0.0002	0.0126		0.4948	−0.0052	3.3453		0.5097	0.0097	0.7602
		MLS	0.9001	0.0001	0.0219		0.5073	0.0073	4.8925		0.5022	0.0022	0.9723
		MM	0.9001	0.0001	0.0219		0.4730	−0.0270	7.3405		0.5296	0.0296	2.2908
		MLM	0.9001	0.0001	0.0219		0.5922	0.0922	11.4040		0.4610	−0.0390	1.7020
	150	MLE	0.8998	−0.0002	0.0037		0.5010	0.0010	2.1560		0.5071	0.0071	0.4934
		MLS	0.9000	0.0000	0.0062		0.5057	0.0057	2.9626		0.5005	0.0005	0.6753
		MM	0.9000	0.0000	0.0062		0.4774	−0.0226	5.3286		0.5235	0.0235	1.5998
		MLM	0.9000	0.0000	0.0062		0.5853	0.0853	8.0350		0.4617	−0.0383	1.3375
0.95	30	MLE	0.9522	0.0022	0.6306		0.4870	−0.0130	13.1526		0.5328	0.0328	2.9722
		MLS	0.9528	0.0028	1.0615		0.5235	0.0235	19.0908		0.5038	0.0038	3.0317
		MM	0.9528	0.0028	1.0615		0.4519	−0.0481	20.7407		0.5667	0.0667	7.1428
		MLM	0.9528	0.0028	1.0615		0.6177	0.1177	35.0848		0.4675	−0.0325	3.8529
	50	MLE	0.9493	−0.0007	0.1061		0.4989	−0.0011	6.7388		0.5185	0.0185	1.6173
		MLS	0.9495	−0.0005	0.1821		0.5210	0.0210	9.6292		0.5004	0.0004	1.8929
		MM	0.9495	−0.0005	0.1821		0.4678	−0.0322	12.6475		0.5449	0.0449	4.2641
		MLM	0.9495	−0.0005	0.1821		0.6093	0.1093	20.9550		0.4624	−0.0376	2.6568
	100	MLE	0.9497	−0.0003	0.0162		0.5060	0.0060	3.5016		0.5092	0.0092	0.7257
		MLS	0.9499	−0.0001	0.0245		0.5155	0.0155	4.7581		0.4999	−0.0001	0.9493
		MM	0.9499	−0.0001	0.0245		0.4854	−0.0146	8.0805		0.5262	0.0262	2.3448
		MLM	0.9499	−0.0001	0.0245		0.6000	0.1000	12.5384		0.4604	−0.0396	1.7649
	150	MLE	0.9498	−0.0002	0.0047		0.5006	0.0006	2.2989		0.5070	0.0070	0.4819
		MLS	0.9497	−0.0003	0.0080		0.5086	0.0086	3.1356		0.5026	0.0026	0.6549
		MM	0.9497	−0.0003	0.0080		0.4903	−0.0097	5.6951		0.5186	0.0186	1.5909
		MLM	0.9497	−0.0003	0.0080		0.5934	0.0934	9.1933		0.4603	−0.0397	1.4338
1.05	30	MLE	1.0501	0.0001	0.6603		0.4976	−0.0024	9.6864		0.5295	0.0295	2.7535
		MLS	1.0480	−0.0020	1.1454		0.5435	0.0435	19.0480		0.5020	0.0020	2.9153
		MM	1.0480	−0.0020	1.1454		0.4727	−0.0273	19.6606		0.5618	0.0618	6.7118
		MLM	1.0480	−0.0020	1.1454		0.6449	0.1449	37.9974		0.4613	−0.0387	3.7214
	50	MLE	1.0499	−0.0001	0.1506		0.4998	−0.0002	7.2030		0.5206	0.0206	1.7516
		MLS	1.0500	0.0000	0.2461		0.5222	0.0222	10.3539		0.5037	0.0037	2.1801
		MM	1.0500	0.0000	0.2461		0.4748	−0.0252	13.4412		0.5436	0.0436	4.1555
		MLM	1.0500	0.0000	0.2461		0.6122	0.1122	21.4527		0.4630	−0.0370	2.5071
	100	MLE	1.0504	0.0004	0.0190		0.4930	−0.0070	3.4884		0.5097	0.0097	0.6671
		MLS	1.0506	0.0006	0.0314		0.5025	0.0025	4.3982		0.5006	0.0006	0.9108
		MM	1.0506	0.0006	0.0314		0.4715	−0.0285	8.0895		0.5274	0.0274	2.1731
		MLM	1.0506	0.0006	0.0314		0.5842	0.0842	10.6968		0.4617	−0.0383	1.5892
	150	MLE	1.0500	0.0000	0.0056		0.4949	−0.0051	2.0959		0.5082	0.0082	0.4612
		MLS	1.0499	−0.0001	0.0107		0.5053	0.0053	3.2664		0.5030	0.0030	0.6055
		MM	1.0499	−0.0001	0.0107		0.4889	−0.0111	5.8912		0.5176	0.0176	1.6345
		MLM	1.0499	−0.0001	0.0107		0.5909	0.0909	9.3357		0.4597	−0.0403	1.4546
1.10	30	MLE	1.1005	0.0005	0.8795		0.4953	−0.0047	11.7155		0.5316	0.0316	2.9118
		MLS	1.1037	0.0037	1.4861		0.5256	0.0256	17.2638		0.5031	0.0031	3.3829
		MM	1.1037	0.0037	1.4861		0.4551	−0.0449	19.2821		0.5621	0.0621	6.3725
		MLM	1.1037	0.0037	1.4861		0.6219	0.1219	34.3621		0.4639	−0.0361	3.6346
	50	MLE	1.1000	0.0000	0.1615		0.5059	0.0059	7.3378		0.5141	0.0141	1.5498
		MLS	1.1001	0.0001	0.2752		0.5297	0.0297	10.8373		0.4961	−0.0039	1.8948
		MM	1.1001	0.0001	0.2752		0.4751	−0.0249	12.9128		0.5407	0.0407	3.9492
		MLM	1.1001	0.0001	0.2752		0.6197	0.1197	23.3610		0.4582	−0.0418	2.8084
	100	MLE	1.0999	−0.0001	0.0189		0.4974	−0.0026	3.1563		0.5112	0.0112	0.7756
		MLS	1.0997	−0.0003	0.0319		0.5095	0.0095	4.6968		0.5038	0.0038	0.9466
		MM	1.0997	−0.0003	0.0319		0.4849	−0.0151	7.8079		0.5256	0.0256	2.2592
		MLM	1.0997	−0.0003	0.0319		0.5957	0.0957	12.1002		0.4619	−0.0381	1.6833
	150	MLE	1.0999	−0.0001	0.0059		0.5042	0.0042	2.1381		0.5048	0.0048	0.4252
		MLS	1.0999	−0.0001	0.0103		0.5115	0.0115	3.4377		0.4997	−0.0003	0.6374
		MM	1.0999	−0.0001	0.0103		0.4901	−0.0099	6.2867		0.5169	0.0169	1.4973
		MLM	1.0999	−0.0001	0.0103		0.5977	0.0977	10.1342		0.4566	−0.0434	1.5425

**Table 4:** The simulated Means, Biases and *n* × MSE values for the MLE, MLS, MM and MLM estimators of the parameters *a*, α and λ, when α=1.

				$\hat{a}$				$\hat{\lambda }$				$\hat{\alpha }$	
*a*	*n*	Method	Mean	Bias	*n* × MSE		Mean	Bias	*n* × MSE		Mean	Bias	*n* × MSE
0.90	30	MLE	0.9010	0.0010	0.1293		0.4848	−0.0152	10.0515		1.0578	0.0578	12.7947
		MLS	0.9017	0.0017	0.2193		0.5173	0.0173	13.9132		1.0021	0.0021	14.4717
		MM	0.9017	0.0017	0.2193		0.4977	−0.0023	15.5070		1.0437	0.0437	14.0296
		MLM	0.9017	0.0017	0.2193		0.5821	0.0821	20.2826		0.9189	−0.0811	13.4991
	50	MLE	0.8999	−0.0001	0.0279		0.4962	−0.0038	7.4478		1.0420	0.0420	7.3976
		MLS	0.9005	0.0005	0.0519		0.5120	0.0120	10.1556		1.0102	0.0102	8.5684
		MM	0.9005	0.0005	0.0519		0.5059	0.0059	11.0708		1.0271	0.0271	8.1213
		MLM	0.9005	0.0005	0.0519		0.5794	0.0794	15.5075		0.9191	−0.0809	9.3453
	100	MLE	0.9000	0.0000	0.0034		0.4985	−0.0015	3.6983		1.0207	0.0207	2.9458
		MLS	0.9001	0.0001	0.0060		0.5059	0.0059	5.2876		1.0057	0.0057	4.0647
		MM	0.9001	0.0001	0.0060		0.5038	0.0038	5.6739		1.0112	0.0112	3.5802
		MLM	0.9001	0.0001	0.0060		0.5736	0.0736	8.9471		0.9119	−0.0881	6.4967
	150	MLE	0.9000	0.0000	0.0010		0.4975	−0.0025	2.1136		1.0143	0.0143	1.8608
		MLS	0.9002	0.0002	0.0017		0.4996	−0.0004	2.8741		1.0021	0.0021	2.4144
		MM	0.9002	0.0002	0.0017		0.4999	−0.0001	3.0032		1.0051	0.0051	2.5506
		MLM	0.9002	0.0002	0.0017		0.5667	0.0667	5.5842		0.9101	−0.0899	5.7094
0.95	30	MLE	0.9491	−0.0009	0.1384		0.5040	0.0040	11.5494		1.0620	0.0620	14.5314
		MLS	0.9476	−0.0024	0.2394		0.5533	0.0533	20.7275		1.0077	0.0077	16.7516
		MM	0.9476	−0.0024	0.2394		0.5368	0.0368	20.8239		1.0432	0.0432	15.7827
		MLM	0.9476	−0.0024	0.2394		0.6235	0.1235	29.9155		0.9161	−0.0839	14.7327
	50	MLE	0.9500	0.0000	0.0323		0.5003	0.0003	7.1557		1.0293	0.0293	6.0053
		MLS	0.9502	0.0002	0.0552		0.5225	0.0225	10.8295		0.9968	−0.0032	8.8009
		MM	0.9502	0.0002	0.0552		0.5112	0.0112	10.9200		1.0186	0.0186	7.3102
		MLM	0.9502	0.0002	0.0552		0.5897	0.0897	16.1381		0.9064	−0.0936	9.7342
	100	MLE	0.9499	−0.0001	0.0033		0.5002	0.0002	3.4586		1.0186	0.0186	3.0031
		MLS	0.9498	−0.0002	0.0062		0.5139	0.0139	5.2283		1.0039	0.0039	3.6547
		MM	0.9498	−0.0002	0.0062		0.5142	0.0142	5.4825		1.0071	0.0071	3.7674
		MLM	0.9498	−0.0002	0.0062		0.5827	0.0827	9.5200		0.9095	−0.0905	6.7580
	150	MLE	0.9500	0.0000	0.0011		0.4996	−0.0004	2.3151		1.0123	0.0123	2.0880
		MLS	0.9501	0.0001	0.0020		0.5048	0.0048	3.2918		1.0013	0.0013	2.7135
		MM	0.9501	0.0001	0.0020		0.5036	0.0036	3.2539		1.0066	0.0066	2.6992
		MLM	0.9501	0.0001	0.0020		0.5725	0.0725	6.4420		0.9093	−0.0907	6.0326
1.05	30	MLE	1.0500	0.0000	0.1712		0.4994	−0.0006	14.0982		1.0624	0.0624	11.6667
		MLS	1.0510	0.0010	0.2874		0.5342	0.0342	22.0731		1.0042	0.0042	14.0411
		MM	1.0510	0.0010	0.2874		0.5130	0.0130	20.9944		1.0447	0.0447	12.6039
		MLM	1.0510	0.0010	0.2874		0.5989	0.0989	27.9994		0.9175	−0.0825	12.5305
	50	MLE	1.0496	−0.0004	0.0400		0.5052	0.0052	7.6634		1.0408	0.0408	7.4367
		MLS	1.0502	0.0002	0.0637		0.5230	0.0230	10.5482		1.0066	0.0066	8.6068
		MM	1.0502	0.0002	0.0637		0.5146	0.0146	10.2297		1.0246	0.0246	8.5140
		MLM	1.0502	0.0002	0.0637		0.5910	0.0910	15.8936		0.9140	−0.0860	9.6941
	100	MLE	1.0501	0.0001	0.0048		0.4961	−0.0039	3.7954		1.0212	0.0212	3.2199
		MLS	1.0500	0.0000	0.0088		0.5122	0.0122	5.6305		1.0023	0.0023	3.8942
		MM	1.0500	0.0000	0.0088		0.5075	0.0075	5.9801		1.0135	0.0135	3.9135
		MLM	1.0500	0.0000	0.0088		0.5784	0.0784	9.6700		0.9124	−0.0876	6.6484
	150	MLE	1.0500	0.0000	0.0013		0.4990	−0.0010	2.3699		1.0149	0.0149	1.8542
		MLS	1.0501	0.0001	0.0022		0.5050	0.0050	3.2776		1.0032	0.0032	2.4563
		MM	1.0501	0.0001	0.0022		0.5022	0.0022	3.4271		1.0098	0.0098	2.2668
		MLM	1.0501	0.0001	0.0022		0.5720	0.0720	6.4415		0.9113	−0.0887	5.5864
1.10	30	MLE	1.0997	−0.0003	0.1947		0.4944	−0.0056	12.6568		1.0720	0.0720	13.7050
		MLS	1.0991	−0.0009	0.3127		0.5346	0.0346	17.9753		1.0134	0.0134	16.8987
		MM	1.0991	−0.0009	0.3127		0.5146	0.0146	18.1320		1.0592	0.0592	14.8263
		MLM	1.0991	−0.0009	0.3127		0.6002	0.1002	25.5466		0.9303	−0.0697	12.8303
	50	MLE	1.1003	0.0003	0.0426		0.4904	−0.0096	6.3634		1.0407	0.0407	6.5906
		MLS	1.1005	0.0005	0.0734		0.5135	0.0135	10.1291		1.0057	0.0057	8.3013
		MM	1.1005	0.0005	0.0734		0.5044	0.0044	10.0618		1.0253	0.0253	7.4875
		MLM	1.1005	0.0005	0.0734		0.5796	0.0796	14.7206		0.9160	−0.0840	8.9003
	100	MLE	1.1000	0.0000	0.0050		0.5057	0.0057	3.7003		1.0156	0.0156	3.1842
		MLS	1.1002	0.0002	0.0094		0.5142	0.0142	5.4640		0.9982	−0.0018	4.3067
		MM	1.1002	0.0002	0.0094		0.5121	0.0121	5.5775		1.0054	0.0054	4.0731
		MLM	1.1002	0.0002	0.0094		0.5824	0.0824	9.6593		0.9063	−0.0937	7.3535
	150	MLE	1.0999	−0.0001	0.0016		0.5040	0.0040	2.3823		1.0119	0.0119	1.9819
		MLS	1.0998	−0.0002	0.0027		0.5137	0.0137	3.4826		1.0020	0.0020	2.6826
		MM	1.0998	−0.0002	0.0027		0.5128	0.0128	3.6805		1.0062	0.0062	2.6669
		MLM	1.0998	−0.0002	0.0027		0.5821	0.0821	7.3641		0.9088	−0.0912	6.0368

**Table 5:** The simulated Means, Biases and *n* × MSE values for the MLE, MLS, MM and MLM estimators of the parameters *a*, α and λ, when α=1.5.

				$\hat{a}$				$\hat{\lambda }$				$\hat{\alpha }$	
*a*	*n*	Method	Mean	Bias	*n* × MSE		Mean	Bias	*n* × MSE		Mean	Bias	*n* × MSE
0.90	30	MLE	0.9005	0.0005	0.0524		0.5019	0.0019	11.1830		1.5579	0.0579	24.1329
		MLS	0.9006	0.0006	0.0995		0.5399	0.0399	18.4300		1.4787	−0.0213	37.2741
		MM	0.9006	0.0006	0.0995		0.5260	0.0260	17.5207		1.5193	0.0193	23.9922
		MLM	0.9006	0.0006	0.0995		0.5932	0.0932	22.9796		1.3518	−0.1482	32.9507
	50	MLE	0.9001	0.0001	0.0113		0.5053	0.0053	6.8493		1.5456	0.0456	14.0863
		MLS	0.8999	−0.0001	0.0218		0.5325	0.0325	12.1371		1.4997	−0.0003	18.7168
		MM	0.8999	−0.0001	0.0218		0.5265	0.0265	11.7692		1.5162	0.0162	14.6065
		MLM	0.8999	−0.0001	0.0218		0.5865	0.0865	15.6682		1.3644	−0.1356	21.2329
	100	MLE	0.9000	0.0000	0.0014		0.4973	−0.0027	3.1802		1.5289	0.0289	6.8488
		MLS	0.9001	0.0001	0.0028		0.5074	0.0074	4.8588		1.5053	0.0053	10.7571
		MM	0.9001	0.0001	0.0028		0.5092	0.0092	5.1761		1.5041	0.0041	7.6287
		MLM	0.9001	0.0001	0.0028		0.5640	0.0640	7.1182		1.3664	−0.1336	15.2179
	150	MLE	0.9001	0.0001	0.0004		0.4979	−0.0021	2.1804		1.5092	0.0092	4.2275
		MLS	0.9001	0.0001	0.0008		0.5027	0.0027	3.1175		1.4940	−0.0060	6.1723
		MM	0.9001	0.0001	0.0008		0.5055	0.0055	3.4395		1.4923	−0.0077	5.0529
		MLM	0.9001	0.0001	0.0008		0.5609	0.0609	5.2521		1.3554	−0.1446	14.3443
0.95	30	MLE	0.9489	−0.0011	0.0656		0.5082	0.0082	12.4920		1.5951	0.0951	28.9125
		MLS	0.9493	−0.0007	0.1058		0.5445	0.0445	19.6036		1.5013	0.0013	32.1834
		MM	0.9493	−0.0007	0.1058		0.5313	0.0313	18.8415		1.5461	0.0461	26.4845
		MLM	0.9493	−0.0007	0.1058		0.5938	0.0938	23.8515		1.3818	−0.1182	28.5391
	50	MLE	0.9499	−0.0001	0.0144		0.5002	0.0002	7.9214		1.5549	0.0549	12.8801
		MLS	0.9502	0.0002	0.0263		0.5213	0.0213	12.4934		1.5069	0.0069	18.9876
		MM	0.9502	0.0002	0.0263		0.5177	0.0177	12.4068		1.5194	0.0194	13.4693
		MLM	0.9502	0.0002	0.0263		0.5739	0.0739	15.4281		1.3737	−0.1263	18.7745
	100	MLE	0.9501	0.0001	0.0016		0.4910	−0.0090	3.3127		1.5355	0.0355	6.9247
		MLS	0.9501	0.0001	0.0028		0.5039	0.0039	4.6658		1.5065	0.0065	9.1575
		MM	0.9501	0.0001	0.0028		0.5020	0.0020	4.8121		1.5154	0.0154	7.3091
		MLM	0.9501	0.0001	0.0028		0.5585	0.0585	6.7268		1.3732	−0.1268	13.9895
	150	MLE	0.9500	0.0000	0.0005		0.5019	0.0019	2.0825		1.5179	0.0179	4.0632
		MLS	0.9500	0.0000	0.0008		0.5076	0.0076	2.9870		1.4968	−0.0032	5.7278
		MM	0.9500	0.0000	0.0008		0.5066	0.0066	3.0436		1.5038	0.0038	4.3917
		MLM	0.9500	0.0000	0.0008		0.5644	0.0644	5.2696		1.3616	−0.1384	13.1286
1.05	30	MLE	1.0502	0.0002	0.0738		0.5002	0.0002	11.7897		1.5814	0.0814	25.5731
		MLS	1.0498	−0.0002	0.1359		0.5420	0.0420	19.4166		1.5072	0.0072	33.2336
		MM	1.0498	−0.0002	0.1359		0.5330	0.0330	18.9608		1.5331	0.0331	24.5938
		MLM	1.0498	−0.0002	0.1359		0.5935	0.0935	24.1369		1.3748	−0.1252	28.0659
	50	MLE	1.0498	−0.0002	0.0177		0.5159	0.0159	7.4399		1.5295	0.0295	13.4886
		MLS	1.0500	0.0000	0.0302		0.5346	0.0346	11.6573		1.4843	−0.0157	18.7570
		MM	1.0500	0.0000	0.0302		0.5350	0.0350	11.5492		1.4917	−0.0083	13.5585
		MLM	1.0500	0.0000	0.0302		0.5925	0.0925	15.8869		1.3473	−0.1527	23.3247
	100	MLE	1.0500	0.0000	0.0019		0.4988	−0.0012	3.2830		1.5270	0.0270	6.3639
		MLS	1.0501	0.0001	0.0033		0.5085	0.0085	4.5018		1.5027	0.0027	9.1314
		MM	1.0501	0.0001	0.0033		0.5104	0.0104	4.8801		1.5037	0.0037	6.7632
		MLM	1.0501	0.0001	0.0033		0.5656	0.0656	6.9689		1.3651	−0.1349	14.7173
	150	MLE	1.0499	−0.0001	0.0006		0.5030	0.0030	2.2228		1.5154	0.0154	4.0225
		MLS	1.0499	−0.0001	0.0010		0.5138	0.0138	3.2125		1.5008	0.0008	5.6962
		MM	1.0499	−0.0001	0.0010		0.5141	0.0141	3.3933		1.5020	0.0020	4.3542
		MLM	1.0499	−0.0001	0.0010		0.5713	0.0713	5.9529		1.3612	−0.1388	13.1834
1.10	30	MLE	1.1005	0.0005	0.0860		0.4961	−0.0039	13.5664		1.6118	0.1118	32.1140
		MLS	1.1007	0.0007	0.1496		0.5355	0.0355	20.7399		1.5226	0.0226	33.0628
		MM	1.1007	0.0007	0.1496		0.5217	0.0217	20.6813		1.5654	0.0654	29.0353
		MLM	1.1007	0.0007	0.1496		0.5845	0.0845	25.1897		1.3982	−0.1018	28.2119
	50	MLE	1.1000	0.0000	0.0172		0.4966	−0.0034	7.0449		1.5525	0.0525	13.9447
		MLS	1.1001	0.0001	0.0278		0.5169	0.0169	9.9515		1.5059	0.0059	17.8926
		MM	1.1001	0.0001	0.0278		0.5173	0.0173	10.2355		1.5104	0.0104	14.4451
		MLM	1.1001	0.0001	0.0278		0.5705	0.0705	13.0638		1.3719	−0.1281	19.8360
	100	MLE	1.1001	0.0001	0.0022		0.4952	−0.0048	3.2381		1.5220	0.0220	5.8683
		MLS	1.1001	0.0001	0.0041		0.5071	0.0071	5.0921		1.4980	−0.0020	8.2509
		MM	1.1001	0.0001	0.0041		0.5087	0.0087	5.3660		1.4991	−0.0009	6.7016
		MLM	1.1001	0.0001	0.0041		0.5638	0.0638	7.4414		1.3609	−0.1391	14.7573
	150	MLE	1.1000	0.0000	0.0007		0.5016	0.0016	2.2137		1.5124	0.0124	3.5950
		MLS	1.1001	0.0001	0.0012		0.5068	0.0068	3.1604		1.4916	−0.0084	4.9886
		MM	1.1001	0.0001	0.0012		0.5076	0.0076	3.4193		1.4953	−0.0047	4.4827
		MLM	1.1001	0.0001	0.0012		0.5638	0.0638	5.3355		1.3565	−0.1435	13.5914

**Table 6:** The simulated Means, Biases and *n* × MSE values for the MLE, MLS, MM and MLM estimators of the parameters *a*, α and λ, when α=2.

				$\hat{a}$				$\hat{\lambda }$				$\hat{\alpha }$	
*a*	*n*	Method	Mean	Bias	*n* × MSE		Mean	Bias	*n* × MSE		Mean	Bias	*n* × MSE
0.90	30	MLE	0.8995	−0.0005	0.0312		0.5149	0.0149	12.5198		2.0791	0.0791	45.8154
		MLS	0.8999	−0.0001	0.0550		0.5464	0.0464	20.8241		1.9744	−0.0256	60.5722
		MM	0.8999	−0.0001	0.0550		0.5397	0.0397	19.4525		2.0090	0.0090	47.7261
		MLM	0.8999	−0.0001	0.0550		0.5908	0.0908	23.1001		1.8075	−0.1925	56.0720
	50	MLE	0.8999	−0.0001	0.0064		0.4973	−0.0027	7.0829		2.0903	0.0903	32.1451
		MLS	0.8998	−0.0002	0.0112		0.5224	0.0224	11.3157		2.0245	0.0245	34.6545
		MM	0.8998	−0.0002	0.0112		0.5218	0.0218	11.8369		2.0398	0.0398	35.8893
		MLM	0.8998	−0.0002	0.0112		0.5680	0.0680	13.9942		1.8530	−0.1470	36.1062
	100	MLE	0.9000	0.0000	0.0008		0.4981	−0.0019	3.4018		2.0434	0.0434	12.7264
		MLS	0.9000	0.0000	0.0015		0.5125	0.0125	5.0733		2.0054	0.0054	17.0684
		MM	0.9000	0.0000	0.0015		0.5237	0.0237	6.6999		1.9879	−0.0121	25.4847
		MLM	0.9000	0.0000	0.0015		0.5600	0.0600	6.8883		1.8305	−0.1695	25.8530
	150	MLE	0.9000	0.0000	0.0002		0.4923	−0.0077	1.9933		2.0342	0.0342	7.6607
		MLS	0.9000	0.0000	0.0004		0.5001	0.0001	2.8267		2.0118	0.0118	10.8052
		MM	0.9000	0.0000	0.0004		0.5130	0.0130	4.5657		1.9858	−0.0142	18.8897
		MLM	0.9000	0.0000	0.0004		0.5499	0.0499	4.1798		1.8302	−0.1698	21.1514
0.95	30	MLE	0.9499	−0.0001	0.0355		0.5031	0.0031	12.3580		2.1123	0.1123	48.9833
		MLS	0.9502	0.0002	0.0551		0.5358	0.0358	17.7030		1.9956	−0.0044	63.7955
		MM	0.9502	0.0002	0.0551		0.5283	0.0283	17.7039		2.0425	0.0425	52.2485
		MLM	0.9502	0.0002	0.0551		0.5789	0.0789	20.3822		1.8373	−0.1627	52.9402
	50	MLE	0.9496	−0.0004	0.0073		0.5028	0.0028	6.8502		2.0839	0.0839	25.3437
		MLS	0.9497	−0.0003	0.0118		0.5247	0.0247	10.3574		2.0115	0.0115	28.7200
		MM	0.9497	−0.0003	0.0118		0.5293	0.0293	10.9572		2.0186	0.0186	34.2805
		MLM	0.9497	−0.0003	0.0118		0.5708	0.0708	12.3001		1.8412	−0.1588	32.0589
	100	MLE	0.9499	−0.0001	0.0010		0.5019	0.0019	3.5575		2.0443	0.0443	13.5813
		MLS	0.9498	−0.0002	0.0017		0.5171	0.0171	4.9603		2.0145	0.0145	17.5450
		MM	0.9498	−0.0002	0.0017		0.5245	0.0245	6.0250		2.0006	0.0006	21.4047
		MLM	0.9498	−0.0002	0.0017		0.5663	0.0663	7.2656		1.8322	−0.1678	25.6449
	150	MLE	0.9500	0.0000	0.0003		0.4990	−0.0010	2.2081		2.0239	0.0239	6.8783
		MLS	0.9500	0.0000	0.0005		0.5050	0.0050	3.1609		1.9992	−0.0008	9.9285
		MM	0.9500	0.0000	0.0005		0.5188	0.0188	4.9578		1.9730	−0.0270	20.2418
		MLM	0.9500	0.0000	0.0005		0.5551	0.0551	4.7154		1.8181	−0.1819	22.9365
1.05	30	MLE	1.0500	0.0000	0.0427		0.4959	−0.0041	11.6756		2.1083	0.1083	42.9917
		MLS	1.0501	0.0001	0.0799		0.5354	0.0354	19.9111		1.9971	−0.0029	54.5672
		MM	1.0501	0.0001	0.0799		0.5253	0.0253	19.6131		2.0403	0.0403	43.2972
		MLM	1.0501	0.0001	0.0799		0.5764	0.0764	22.4154		1.8360	−0.1640	46.8632
	50	MLE	1.0499	−0.0001	0.0086		0.5003	0.0003	6.2178		2.0690	0.0690	23.5081
		MLS	1.0497	−0.0003	0.0159		0.5264	0.0264	10.3455		2.0061	0.0061	30.6029
		MM	1.0497	−0.0003	0.0159		0.5297	0.0297	10.8398		2.0094	0.0094	30.3326
		MLM	1.0497	−0.0003	0.0159		0.5740	0.0740	13.1906		1.8294	−0.1706	34.2805
	100	MLE	1.0499	−0.0001	0.0011		0.5050	0.0050	3.2890		2.0371	0.0371	11.3522
		MLS	1.0498	−0.0002	0.0018		0.5200	0.0200	4.9662		2.0028	0.0028	15.2171
		MM	1.0498	−0.0002	0.0018		0.5316	0.0316	6.6579		1.9862	−0.0138	22.6680
		MLM	1.0498	−0.0002	0.0018		0.5700	0.0700	7.4521		1.8234	−0.1766	25.2576
	150	MLE	1.0499	−0.0001	0.0004		0.5015	0.0015	2.4090		2.0301	0.0301	7.0388
		MLS	1.0500	0.0000	0.0006		0.5070	0.0070	3.3655		2.0083	0.0083	10.3245
		MM	1.0500	0.0000	0.0006		0.5236	0.0236	5.4584		1.9740	−0.0260	21.4168
		MLM	1.0500	0.0000	0.0006		0.5576	0.0576	5.1566		1.8241	−0.1759	21.9860
1.10	30	MLE	1.1001	0.0001	0.0488		0.5005	0.0005	12.1285		2.1189	0.1189	49.2894
		MLS	1.1004	0.0004	0.0847		0.5350	0.0350	19.0533		2.0038	0.0038	55.6672
		MM	1.1004	0.0004	0.0847		0.5292	0.0292	18.7982		2.0436	0.0436	53.1739
		MLM	1.1004	0.0004	0.0847		0.5772	0.0772	21.3407		1.8439	−0.1561	50.4119
	50	MLE	1.0998	−0.0002	0.0091		0.4960	−0.0040	6.7759		2.0861	0.0861	28.3994
		MLS	1.0997	−0.0003	0.0152		0.5195	0.0195	9.5662		2.0217	0.0217	35.9142
		MM	1.0997	−0.0003	0.0152		0.5272	0.0272	10.9293		2.0163	0.0163	39.2754
		MLM	1.0997	−0.0003	0.0152		0.5661	0.0661	11.8486		1.8456	−0.1544	35.1339
	100	MLE	1.1001	0.0001	0.0011		0.4940	−0.0060	3.2518		2.0434	0.0434	10.8650
		MLS	1.1001	0.0001	0.0023		0.5049	0.0049	5.4451		2.0098	0.0098	15.9720
		MM	1.1001	0.0001	0.0023		0.5142	0.0142	6.2465		1.9936	−0.0064	19.3889
		MLM	1.1001	0.0001	0.0023		0.5547	0.0547	6.9994		1.8281	−0.1719	24.0368
	150	MLE	1.1000	0.0000	0.0004		0.4960	−0.0040	2.3576		2.0257	0.0257	6.9247
		MLS	1.1001	0.0001	0.0007		0.5022	0.0022	3.3998		2.0010	0.0010	9.3611
		MM	1.1001	0.0001	0.0007		0.5197	0.0197	5.7844		1.9680	−0.0320	22.4736
		MLM	1.1001	0.0001	0.0007		0.5524	0.0524	4.9670		1.8206	−0.1794	22.3078

**Table 7:** Estimation of parameters for the Aircraft data set and the process comparison.

Method		$\hat{a}$		$\hat{\lambda }$		$\hat{\alpha }$		MSE		MPE
MLE		1.043953		0.651392		0.105436		4.3955 × 10^3^		0.2904
MLS				0.564054		0.147776		4.4400 × 10^3^		0.3515
MM		1.050087		0.622486		0.120725		4.3981 × 10^3^		0.2825
MLM				0.597786		0.137541		4.4038 × 10^3^		0.2907
RP		1.000000		0.630965		0.163402		4.9956 × 10^3^		0.5848

**Table 8:** AIC and negative Log-Likelihood values for the Aircraft data set.

	Model			
	Power Lindley	Log-Normal	Gamma	Inverse Gaussian
AIC	306.4221	307.9897	557.08	307.6517
-L	150.2111	150.9812	275.54	150.8258

**Table 9:** Estimation of parameters for the coal mining disaster data and process comparison.

Method		$\hat{a}$		$\hat{\lambda }$		$\hat{\alpha }$		MSE		MPE
MLE		0.990772		0.650397		0.127163		8.1662 × 10^4^		0.5102
MLS				0.647176		0.126658		8.1680 × 10^4^		0.4949
MM		0.990912		0.620836		0.146652		8.1975 × 10^4^		0.5138
MLM				0.626483		0.140979		8.1789 × 10^4^		0.5031
RP		1.000000		0.576175		0.103469		9.7808 × 10^4^		2.3976

**Table 10:** AIC and negative Log-Likelihood values for coal mining disaster data.

	Model			
	Power Lindley	Log-Normal	Gamma	Inverse Gaussian
AIC	2365	2426	2366	2517
-L	11785	11928	11787	12528

## Appendix A

According to [26], the first two population L-moments of a continuous distribution are calculated by

(A1) $$L_{1}=\int_{-\infty }^{\infty }xf\left(x\right)dx$$

and

(A2) $$L_{2}=\int_{-\infty }^{\infty }x\left(2F\left(x\right)-1\right)f\left(x\right)dx,$$

respectively, where $f\left(x\right)$ and $F\left(x\right)$ are the pdf and the cdf of the random variable *X*. Obviously, the first popuplation L-moment $L_{1}$ is the expectation of random variable X. Thus, from Table 2, the first L-moment of Power Lindley distribution is

(A3) $$L_{1}=\frac{\Gamma \left(\frac{1}{\alpha }\right)\left[\alpha \left(\lambda +1\right)+1\right]}{\alpha^{2}\lambda^{1/\alpha }\left(\lambda +1\right)}.$$

Now, we calculate the second population L-moment of Power Lindley distribution. Substituting the cdf given by (5) into (A2), we can write

$$\begin{array}{cc} L_{2} & =\int_{0}^{\infty }x\left(2\left(1-\left(1+\frac{\lambda }{\lambda +1}x^{\alpha }\right)e^{-\lambda x^{\alpha }}\right)-1\right)f\left(x\right)dx\\ & =\int_{0}^{\infty }x\left(2\left(1-\left(1+\frac{\lambda }{\lambda +1}x^{\alpha }\right)e^{-\lambda x^{\alpha }}\right)-1\right)f\left(x\right)dx\\ & =\int_{0}^{\infty }\left(2x\left(1-\left(1+\frac{\lambda }{\lambda +1}x^{\alpha }\right)e^{-\lambda x^{\alpha }}\right)-x\right)f\left(x\right)dx\\ & =\int_{0}^{\infty }2x\left(1-\left(1+\frac{\lambda }{\lambda +1}x^{\alpha }\right)e^{-\lambda x^{\alpha }}\right)f\left(x\right)dx-\int_{0}^{\infty }xf\left(x\right)dx\\ & =\int_{0}^{\infty }2xf\left(x\right)dx-\int_{0}^{\infty }2x\left(1+\frac{\lambda }{\lambda +1}x^{\alpha }\right)e^{-\lambda x^{\alpha }}f\left(x\right)dx-\int_{0}^{\infty }xf\left(x\right)dx\\ & =\int_{0}^{\infty }xf\left(x\right)dx-\int_{0}^{\infty }2x\left(1+\frac{\lambda }{\lambda +1}x^{\alpha }\right)e^{-\lambda x^{\alpha }}f\left(x\right)dx\\ & =\int_{0}^{\infty }xf\left(x\right)dx-\int_{0}^{\infty }2x\left(\frac{\lambda +1+\lambda x^{\alpha }}{\lambda +1}\right)e^{-\lambda x^{\alpha }}f\left(x\right)dx\\ & =E\left(X\right)-\int_{0}^{\infty }2x\left(\frac{\lambda +1+\lambda x^{\alpha }}{\lambda +1}\right)e^{-\lambda x^{\alpha }}f\left(x\right)dx\\ & =\frac{\Gamma \left(\frac{1}{\alpha }\right)\left[\alpha \left(\lambda +1\right)+1\right]}{\alpha^{2}\lambda^{1/\alpha }\left(\lambda +1\right)}-\int_{0}^{\infty }2x\left(1+\frac{\lambda x^{\alpha }}{\lambda +1}\right)e^{-\lambda x^{\alpha }}f\left(x\right)dx\\ & =\frac{\Gamma \left(\frac{1}{\alpha }\right)\left[\alpha \left(\lambda +1\right)+1\right]}{\alpha^{2}\lambda^{1/\alpha }\left(\lambda +1\right)}-\int_{0}^{\infty }2xe^{-\lambda x^{\alpha }}f\left(x\right)dx-\int_{0}^{\infty }2\frac{\lambda x^{\alpha +1}}{\lambda +1}e^{-\lambda x^{\alpha }}f\left(x\right)dx. \end{array}$$

In the last equality, using $f\left(x\right)$ given in (4), it can be easily written

(A4) $$\begin{array}{cc} L_{2} & =\left\{\frac{\Gamma \left(\frac{1}{\alpha }\right)\left[\alpha \left(\lambda +1\right)+1\right]}{\alpha^{2}\lambda^{1/\alpha }\left(\lambda +1\right)}-\int_{0}^{\infty }2xe^{-\lambda x^{\alpha }}\frac{\alpha \lambda^{2}}{1+\lambda }\left(1+x^{\alpha }\right)x^{\alpha -1}e^{-\lambda x^{\alpha }}dx-\right.\\ & \;\;\;\;\;\;\;\;\;\left.\int_{0}^{\infty }2\frac{\lambda x^{\alpha +1}}{\lambda +1}e^{-\lambda x^{\alpha }}\frac{\alpha \lambda^{2}}{1+\lambda }\left(1+x^{\alpha }\right)x^{\alpha -1}e^{-\lambda x^{\alpha }}dx\right\}\\ & =\left\{\frac{\Gamma \left(\frac{1}{\alpha }\right)\left[\alpha \left(\lambda +1\right)+1\right]}{\alpha^{2}\lambda^{1/\alpha }\left(\lambda +1\right)}-2\int_{0}^{\infty }\frac{\alpha \lambda^{2}}{1+\lambda }\left(1+x^{\alpha }\right)x^{\alpha }e^{-2\lambda x^{\alpha }}dx-\right.\\ & \;\;\;\;\;\;\;\;\;\left.\frac{2\lambda }{1+\lambda }\int_{0}^{\infty }\frac{\alpha \lambda^{2}}{1+\lambda }\left(1+x^{\alpha }\right)x^{2\alpha }e^{-2\lambda x^{\alpha }}dx\right\}\\ & =\left\{\frac{\Gamma \left(\frac{1}{\alpha }\right)\left[\alpha \left(\lambda +1\right)+1\right]}{\alpha^{2}\lambda^{1/\alpha }\left(\lambda +1\right)}-\frac{2\alpha \lambda^{2}}{1+\lambda }\int_{0}^{\infty }\left(x^{\alpha }+x^{2\alpha }\right)e^{-2\lambda x^{\alpha }}dx-\right.\\ & \;\;\;\;\;\;\;\;\;\left.\frac{2\alpha \lambda^{3}}{{\left(1+\lambda \right)}^{2}}\int_{0}^{\infty }\left(x^{2\alpha }+x^{3\alpha }\right)e^{-2\lambda x^{\alpha }}dx\right\}\\ & =\left\{\frac{\Gamma \left(\frac{1}{\alpha }\right)\left[\alpha \left(\lambda +1\right)+1\right]}{\alpha^{2}\lambda^{1/\alpha }\left(\lambda +1\right)}-\frac{2\alpha \lambda^{2}}{1+\lambda }\left[\int_{0}^{\infty }x^{\alpha }e^{-2\lambda x^{\alpha }}dx+\int_{0}^{\infty }x^{2\alpha }e^{-2\lambda x^{\alpha }}dx\right]-\right.\\ & \;\;\;\;\;\;\;\left.\;\frac{2\alpha \lambda^{3}}{{\left(1+\lambda \right)}^{2}}\left[\int_{0}^{\infty }x^{2\alpha }e^{-2\lambda x^{\alpha }}dx+\int_{0}^{\infty }x^{3\alpha }e^{-2\lambda x^{\alpha }}dx\right]\right\}. \end{array}$$

Therefore, by using the gamma function, we have

(A5) $$\begin{array}{cc} L_{2} & =\left\{\frac{\Gamma \left(\frac{1}{\alpha }\right)\left(\alpha \left(\lambda +1\right)+1\right)}{\alpha^{2}\lambda^{\frac{1}{\alpha }}\left(\lambda +1\right)}-\frac{2\alpha \lambda^{2}}{1+\lambda }\left(\frac{\Gamma \left(\frac{\alpha +1}{\alpha }\right)}{\alpha {\left(2\lambda \right)}^{\frac{\alpha +1}{\alpha }}}+\frac{\Gamma \left(\frac{2\alpha +1}{\alpha }\right)}{\alpha {\left(2\lambda \right)}^{\frac{2\alpha +1}{\alpha }}}\right)-\right.\;\\ & \;\;\;\left.\frac{2\alpha \lambda^{3}}{{\left(1+\lambda \right)}^{2}}\left(\frac{\Gamma \left(\frac{2\alpha +1}{\alpha }\right)}{\alpha {\left(2\lambda \right)}^{\frac{2\alpha +1}{\alpha }}}+\frac{\Gamma \left(\frac{3\alpha +1}{\alpha }\right)}{\alpha {\left(2\lambda \right)}^{\frac{3\alpha +1}{\alpha }}}\right)\right\}. \end{array}$$
